# Significant differences in the use of healthcare resources of native-born and foreign born in Spain

**DOI:** 10.1186/1471-2458-9-201

**Published:** 2009-06-25

**Authors:** Pilar Carrasco-Garrido, Rodrigo Jiménez-García, Valentin Hernández Barrera, Ana López de Andrés, Ángel Gil de Miguel

**Affiliations:** 1 Unit of Preventive Medicine and Public Health, Rey Juan Carlos University, Alcorcón, Madrid, Spain

## Abstract

**Background:**

In the last decade, the number of foreign residents in Spain has doubled and it has become one of the countries in the European Union with the highest number of immigrants There is no doubt that the health of the immigrant population has become a relevant subject from the point of view of public healthcare. Our study aimed at describing the potential inequalities in the use of healthcare resources and in the lifestyles of the resident immigrant population of Spain.

**Methods:**

Cross-sectional, epidemiological study from the Spanish National Health Survey (NHS) in 2006, from the Ministry of Health and Consumer Affairs. We have worked with individualized secondary data, collected in the Spanish National Health Survey carried out in 2006 and 2007 (SNHS-06), from the Ministry of Health and Consumer Affairs. The format of the SNHS-06 has been adapted to the requirements of the European project for the carrying out of health surveys.

**Results:**

The economic immigrant population resident in Spain, present diseases that are similar to those of the indigenous population. The immigrant population shows significantly lower values in the consumption of alcohol, tobacco and physical activity (OR = 0.76; CI 95%: 0.65–0.89, they nonetheless perceive their health condition as worse than that reported by the autochthonous population (OR = 1.63, CI 95%: 1.34–1.97). The probability of the immigrant population using emergency services in the last 12 months was significantly greater than that of the autochthonous population (OR = 1.31, CI 95%: 1.12–1.54). This situation repeats itself when analyzing hospitalization data, with values of probability of being hospitalized greater among immigrants (OR = 1.39, CI 95%: 1.07–1.81).

**Conclusion:**

The economic immigrants have better parameters in relation to lifestyles, but they have a poor perception of their health. Despite the fact that immigrant population shows higher percentages of emergency attendance and hospitalization than the indigenous population, with respect to the use of healthcare resources, their usage of healthcare resources such as drugs, influenza vaccinations or visits to the dentist is lower.

## Background

Significant growth in immigration in Spain occurred at the end of the nineties [1]. Currently, the Spanish population has grown in excess of 46 million people of which, nearly 11.3% are foreigners, although this percentage does not reflect the population without legal status [2]. In the last decade, the number of foreign residents in Spain has doubled and it has become one of the countries in the European Union with the highest number of immigrants [3]. In the year 2007 the National Immigrant Survey was performed for the first time in our country and it indicated that there are 2.15 million homes in which at least one of the adult members was born outside of Spain, highlighting the fundamentally economic nature of immigration to Spain [4], meaning immigrants from countries more economically disadvantaged than our own. These migratory flows frequently originate from countries with very different healthcare conditions [5-9].

There is no doubt that the health of the immigrant population has become a relevant subject from the point of view of public healthcare [10,11]. Studies on the inequalities in health have been the subject of vast quantities of research within the last decade, countries with significant differences with respect to the healthcare model such as the United States, Canada and some countries of the European Union, have incorporated data regarding knowledge of the healthcare determinants and needs of their immigrant population into their respective National Health Surveys [12-15]. Although different studies on the health of the immigrant population have been made in Spain, nonetheless, in recent years investigations are being carried out that broach the subject of the inequalities in the use of healthcare services by this population group [8,16-18], as the Spanish National Health System attends both legal and illegal immigrants. Theoretically a valid identity documents (healthcare card) is necessary to be attended yet the truth is that immigrants who lack this document and remain in the country as illegal aliens are also attended by the health system. It is important to note that in order to receive the healthcare card the only requisite is a registration certificate from the municipal register. That in order to register in the municipal register there is theoretically no need to be legal, people only need to show an ID but not a residence permit; and that in absence of a healthcare card, the only healthcare service that attends people are the EDs, primary care access is restricted in most cases to people with healthcare card.

In the year 2003, the Spanish National Health Survey (SNHS) [19] included data from the immigrant population residing in the country for the first time in consideration of the new socio-demographic conditions that are present. The gathering of this information continued and was broadened in the latest Spanish National Health Survey for 2006 (SNHS-06) [20]. Even more during this period, specifically in year 2004, there was a Regularization Process in Spain and around 800.000 immigrants previously considered illegal, received residence cards [21].

In this context and following along the lines of the first national health survey of immigrants, we propose, as the objective of our study, to describe the potential inequalities in the use of healthcare resources and in the lifestyles of the resident immigrant population of Spain, identifying the differences between that population and the autochthonous population.

## Methods

A descriptive, cross-sectional epidemiological study has been performed regarding the health profiles, lifestyles and use of healthcare resources by the resident immigrant population of Spain.

We have worked with individualized secondary data, collected in the Spanish National Health Survey carried out in 2006 and 2007 (SNHS-06) [20], from the Ministry of Health and Consumer Affairs. The format of the SNHS-06 has been adapted to the requirements of the European project for the carrying out of health surveys [22].

This survey was performed on a wide sample of the non-institutionalized resident population of Spain, using direct interviews. The sampling procedure is polystage and stratified. The first stage sample units are the census sections and the second stage units are the primary family residences. One adult from each home is selected by random route methods and gender and age quotas to complete the questionnaire. This survey includes data from 29,478 adults. Details on the methodology are described elsewhere [20].

Questions like "*What is your nationality?*" and "*What is the country of your nationality?*" were used to define the economic immigrant variable. Based on these answers, individuals whose nationality did not correspond to the European Union (EU), United States (USA) or Canada, were defined as *economic immigrants*. This included individuals from both sexes, 16 or older, residing in family houses in Spain when the survey took place. The autochthonous population of the study included individuals who were born in Spain and individuals whose nationality corresponded to a country within the EU, the USA or Canada (Spaniard and not economic immigrants). Independent variables collected in the study are the primary socio-demographic characteristics of the population such as age, gender, marital status, level of education and employment situation including the total monthly income in Euros at home.

Variables related to illnesses which the subjects suffer from (comorbidity), collected by way of the question *Has the doctor told you that you suffer from one of these diseases?*, including pathologies such as arterial hypertension, hypercholesterolemia, heart disease, diabetes, asthma, gastric or duodenal ulcers, depression and anxiety, bronchitis, allergies, obesity, arthrosis, and back pain were also analyzed. Variables related to lifestyle used in the study were smoking habits (current smokers and non-smokers, ex-smokers) and consumption of any alcoholic beverages in the two weeks prior to the survey (both defined as dichotomous variables) or leisure time physical activity (moderate, light or no physical activity). Self-rated health by the population was also asked about as a dichotomous variable (very good and good/normal, poor and very poor).

In order to assess the use of healthcare resources, subjects were asked about visits to the doctor (time elapsed since the last visit), visits to the dentist in the last 3 months, whether they had been hospitalized (with hospitalization understood as at least one night in the last 12 months), cause of admissions, and the use of emergency services in the last 12 months (dichotomous variables: yes/no).

They were also asked if they had taken any type of medication in the last two weeks (dichotomous variable "yes", "no") and if they had been self-medicated (understanding as such, consumption of these medications without prescription) or if they had resorted to the use of alternative medicine. The population was also asked if they had received an influenza vaccine in the last vaccination campaign (vaccinated against influenza or not).

Finally, and for the identification of the use of specific resources by the female population of the study, they were asked whether they had ever been to a gynecologist, (affirmative or negative responses).

### Statistical analysis

A descriptive statistic has been made from the primary variables included in the study. The corresponding frequency distributions of the qualitative variables have been calculated, analyzing whether significant differences exist between the two study populations (economic immigrants and autochthonous people). For the bivariate comparison of proportions, the Pearson χ^2 ^method or the Fisher exact test method was applied, with values of p < 0.05 considered significant.

In the analysis of comorbidity, lifestyles and use of healthcare resources, in order to estimate the independent effect of each one of these variables on the study populations (economic immigrants and autochthonous population), the adjusted odds ratios (OR) with their corresponding confidence intervals of 95% (CI 95%) were calculated through multivariate analysis, using logistic regression models to do so. Variables which showed significant association in the bivariate analysis and the adjustment variables considered relevant in scientific literature were included in these multivariate models.

Estimates were made by incorporating the sample weights, through the use of the "svy" (survey command) functions of the STATA program, which allow us to incorporate the sampling design into all our statistical calculations (descriptive, Chi-squared, logistic regression).

## Results

We began with a sample made up of 28,042 autochthonous subjects and a total of 1,436 non-community, economic immigrants, residing in Spain. In regard to the origin of this foreign population, 64.5% came from Latin America, 22.1% from the African continent, 9.3% from European countries and 4.1% from Asia. Within the autochthonous population (Spaniard and not economic immigrants), 2.13% were from the European community and 0.07% were American and Canadian. We compare the age distribution, sex and origin of immigrants from the SNHS 06 with the immigrant population of the National Immigrant Survey 2007 (table 1) On Table 2, the distribution of the different socio-demographic characteristics of both populations is described. Statistically significant differences appear in the level of education, marital status and occupation where 69.4% of immigrants are actively employed.

**Table 1:** Distribution, sex and country of origin of immigrants from the Spanish National Health Survey 2006 with the immigrant population of the Spanish National Immigrant Survey 2007 and the Municipal Register of the National Statistics Institute 2007.

	**SNHS 06**	**SNIS 07**	NSI 07*
	**IMMIGRANT POPULATION OF STUDY**	**IMMIGRANT POPULATION**	**IMMIGRANT POPULATION**
**Sex**			
Men	46.96%	53%	53%
Women	53.04%	47%	47%
Age group (*)			
16–24	18.50%	14,91%	14,7%
25–34	37.95%	31,03%	29.5%
35–44	25.25%	26,33%	20.5%
45–54	12.06%	13,12%	10.4%
55–64	4.43%	7,30%	5.7%
65–74	1.15%	4,57%	3.4%
75 and over	0.66%	2,70%	1.5%
**Country of origin**			
Development countries without EU_25 and without Spain.	0.07%	14.3%	4.6%
EU_25 without Spain	2.1%	21.5%	37.8%**
Countries of Latino America	64.5%	39.4%	34.2%
Countries of Africa	22.1%	16.9	17.8%
Countries of Asia	4.1%	7.6	4.8%
Others	7,1%	0.3	0.8%

National Statistics Institute (NSI). Municipal Register: statistical use and List of place names.

** EU_27

**Table 2:** Distribution of the study population by country of origin. Sociodemographic variables. Spanish National Health Survey 2006.

	**ORIGIN**			
	
	**ECONOMIC IMMIGRANT**		**AUTOCHTHONOUS POPULATION (SPANIARDS AND NOT ECONOMIC IMMIGRANTS)**	
	**%**	**CI 95%**	**%**	**CI 95%**

**Sex**				

Men	46.96	43.40–50.56	49.23	48.42–50.05

Women	53.04	49.44–56.60	50.77	49.95–51.58

Age group (*)				

16–24	18.50	15.73–21.64	11.93	11.33–12.55

25–34	37.95	34.54–41.49	18.81	18.12–19.52

35–44	25.25	22.40–28.34	18.87	18.27–19.47

45–54	12.06	10–14.47	16.11	15.54–16.71

55–64	4.43	3.13–6.22	13.36	12.84–13.89

65–74	1.15	0.64–2.05	11.07	10.63–11.53

75 and over	0.66	0.35–1.23	9.85	9.44–10.28

Marital status (*)				

Single/Divorced/widow	31.40	28.10–34.89	35.66	34.86–36.46

Married or living together	68.60	65.11–71.90	64.34	63.54–65.14

Educational level (*)				

No formal education	6.85	5.13–9.11	11.69	11.22–12.18

Junior school	39.38	35.95–42.32	43.78	42.98–44.58

High school	38.19	34.78–41.73	27.40	26.65–28.16

University and higher education	15.57	13.25–18.22	17.13	16.51–17.77

Occupational status(*)				

Employed	69.39	66.01–72.57	49.81	49–50.62

Unemployed	9.77	7.84–12.12	6.92	6.50–7.37

Inactive	20.84	18.10–23.88	43.27	42.48–44.06

Size of town(*)				

Rural (≤ 10,000 inhabitants)	10.63	8.79–12.80	22.59	21.94–23.25

Urban(>10,000 inhabitants)	89.37	87.20–91.21	77.41	76.75–78.06

**Monthly income**				

≤ 1200 euros	44.85	41.20–48.56	43.14	42.30–44

> 1200 euros	55.15	51.44–58.80	56.86	56–57.70

(*) Statistically significant differences (p < 0.05)

Prevalence of illnesses reported both in the immigrant and autochthonous populations, with the odds ratio adjusted for age and sex and their corresponding confidence intervals of 95%, are shown on Table 3. We observed that the immigrant population shows significantly lower values in pathologies such as hypercholesterolemia, heart diseases and depression and anxiety, however the probability of suffering from gastrointestinal ulcers is practically double among immigrants (OR = 2.09, CI 95%: 1.54–2.83).

**Table 3:** Prevalence of self-reported diseases in immigrant and autochthonous subjects included in the Spanish National Health Survey 2006.

**ORIGIN**						
	**ECONOMIC IMMIGRANT**		**AUTOCHTHONOUS POPULATION (SPANIARDS AND NOT ECONOMIC IMMIGRANTS)**			

	**%**	**CI 95%**	**%**	**CI 95%**	**OR**	**CI 95%**

**High blood pressure**	8.69	6.83–11.0	21.64.	21.02–22.28	0.80	0.59–1.37


**Hearth disease**	2.11	1.40–3.15	7.73	7.35–8.14	0.59	0.39–0.91

**High cholesterol**	5.20	3.97–6.78	16.66	16.10–17.24	0.50	0.37–0.67

**Diabetes**	2.30	1.42–.371	6.49	6.13–6.88	0.85	0.51–1.37

**Asthma**	3.44	2.44–4.85	5.63	5.27–6.01	0.56	0.38–0.81

**Bronchitis**	2.12	1.36–3.30	4.99	4.66–5.35	0.63	0.40–1.00

**Allergy**	10.17	8.23–12.51	12.28	11.76–12.83	0.68	0.53–0.86

**Gastric ulcers**	6.15	4.68–8.05	5.54	5.20–5.90	2.09	1.54–2.83

**Arthrosis**	7.15	5.55–9.17	21.67	21.06–22.29	0.65	0.49–0.87

**Depression, anxiety**	7.22	5.66–9.18	14.31	13.79–14.85	0.66	0.50–0.86

**Migraine**	11.86	9.77–14.31	11.94	11.45–12.45	1.06	0.84–1.34

**Backpain**	19.97	17.34–22.89	29.16	28.45–29.87	0.82	0.68–0.99

**Obesity**						

BMI > 30	11.28	9.21–13.74	15.34	14.75–15.96	1.02	0.81–1.30

Comparison of prevalence using the calculation of adjusted odds ratios using autochthonous subjects as a reference category.

Odds ratio (OR) adjusted by age and sex.

When lifestyles and self-rated health in both groups are analyzed (Table 4), even though the immigrant population shows significantly lower values in the consumption of alcohol and tobacco. As for physical activity, immigrants register significantly lower level (OR = 0.76; CI 95%: 0.65–0.89); they nonetheless perceive their health condition as worse than that reported by the Spanish population (OR = 1.63, CI 95%: 1.34–1.97).

**Table 4:** Prevalence of self-rated health, alcohol consumption, smoking habits and physical exercise in immigrant and autochthonous subjects included in the Spanish National Health Survey 2006; Comparison of prevalence using the calculation of adjusted odds ratios using autochthonous subjects as a reference category.

**ORIGIN**						
	**ECONOMIC IMMIGRANT**		**AUTOCHTHONOUS POPULATION (SPANIARDS AND NOT ECONOMIC IMMIGRANTS)**			

	**%**	**CI 95%**	**%**	**CI 95%**	**OR**	**CI 95%**

**Self-assessment of health status**						

Fair/Poor/Very poor	29.24	26.05–32.64	33.84	33.10–34.59	1.63	1.34–1.97

**Alcohol consumption**	44.71	41.18–48.30	56.69	55.89–57.48	0.56	0.48–0.65

**Smoking habit**	27.73	24.58–31.13	29.65	28.89–30.42	0.71	0.60–0.85

**Physical exercise**	53.27	49.71–56.80	59.59	58.79–60.38	0.76	0.65–0.89

Odds ratio (OR) adjusted by age, sex and co morbidity.

When analyzing the probability of differences in the use of healthcare resources (Table 5), we observed that the probability of the immigrant population using emergency services in the last 12 months, was significantly greater than that of the autochthonous population (OR = 1.31, CI 95%: 1.12–1.54). This situation repeats itself when analyzing hospitalization data, with values of probability of being hospitalized greater among immigrants (OR = 1.39, CI 95%: 1.07–1.81). When hospital admittance is analyzed, the most common among immigrants were deliveries (30.8% CI 95%: [21.4–42.1] in women immigrants vs. autochthonous females 12.6% CI95%: 11.1–14.4]), followed by surgery (29.9% CI 95%: [20.8–41.0] vs. 45% CI 95%: [42.5–47.5] of the autochthonous population).

**Table 5:** Frequency of use of healthcare resources in immigrant and autochthonous subjects included in the Spanish National Health Survey 2006.

**ORIGIN**						
	**ECONOMIC IMMIGRANT**		**AUTOCHTHONOUS POPULATION (SPANIARDS AND NOT ECONOMIC IMMIGRANTS)**			

	**%**	**CI 95%**	**%**	**CI 95%**	**OR**	**CI 95%**

**Medical consultation**	29.73	26.61–33.07	39.11	38.34–39.11	0.93	0.78–1.09

**Hospitalization in preceding 12 months**	10.52	8.38–13.12	9.51	9.06–9.98	1.39	1.07–1.81

**Emergency visit in preceding 12 months**	36.04	32.66–39.56	28.95	28.22–29.69	1.31	1.12–1.54

**Dental visit in preceding 3 months**	14.84	12.63–17.36	17.71	17.10–18.34	0.73	0.60–0.89

**Medications consumption**	54.08	50.50–57.61	66.91	66.12–67.69	0.91	0.77–1.08

**Alternative medicines**	5.37	4.03–7.11	4.83	4.52–5.16	1.38	1.01–1.88

**Self-medication**	18.81	16.19–21.76	15.45	14.86–16.06	1.04	0.86–1.26

**Influenza vaccination**	7.88	6.33–9.77	23.74	23.09–24.40	0.61	0.47–0.79

**Gynecologist visits**	46.69	42.13–51.30	40.03	39.03–41.04	1.18	0.97–1.44

Comparison of frequencies using the calculation of adjusted odds ratios using autochthonous subjects as a reference category.

Odds ratio (OR) adjusted by age, sex and co morbidity.

In regard to practicing preventive medicine, the immigrant population shows values significantly lower than the autochthonous population in visits to the dentist (OR = 0.73, CI 95%: 0.60–0.89) and less probability of receiving vaccination against influenza (OR = 0.61, CI 95%: 0.47–0.79).

Finally, it is notable that although statistically significant differences were not found in the consumption of medications, prescribed as well as auto-medicated, the use of alternative medicines was significantly associated with the immigrant population.

## Discussion

In the year 2003, the Spanish National Health Survey (SNHS) included data from the immigrant population residing in the country for the first time. The two surveys (SNHS03 and SNHS 06) have significant differences the most relevant ones are: 1) Sample size NHS 2003 included 21650 adults and the NHS 2006 29,478 adults, 2) the percentage of immigrants in the NHS 2003 was 2.3% (n = 502) this percentage has risen to 4.9% (n = 1436) in 2006, 3) the immigrants' country of origin has also changed. The most relevant change is a significant increase in the proportion of immigrants coming from Latin America (56% in 2003 to 64.5% in 2006) that reflects more adequately the real immigrant population in Spain when compared with other official sources.

Our results show that economic immigrants residing in Spain during the years 2006 and 2007 are a young population [2,5] and have similar socio-demographic characteristics to the Spanish population and coincide, with regard to the origin, with data supplied by the Spanish National Immigrant Survey [4] although it is true that they have lower economic incomes, as also occurs in other developed nations [23].

According to the results obtained with the SNHS-06 regarding comorbidity in both populations, immigrant individuals declare that they suffer diseases similar to those suffered by the autochthonous population [24,25], some with values that are significantly lower such as with heart disease, diabetes and hypercholesterolemia, coinciding with the results from other studies [26]. Nonetheless, the probability of suffering from gastroduodenal ulcers is greater among immigrants coinciding with the data in a study performed in the Netherlands, the objective of which was to determine the prevalence of and the risk factors for gastroduodenal ulcers in a community of immigrants residing in that country [27].

In our context, we can relate these better health conditions with what is known as the healthy immigrant effect [28,29] as these people have a better health condition when beginning the migratory process and have not spent many years in Spain [4]. However, we must bear in mind that social and economic differences increase vulnerability against health problems, while limiting access to and the use of healthcare resources. Therefore, they may not have had the same probability of being diagnosed.

The variables related to lifestyles show a significantly lower consumption of alcohol and tobacco by the immigrant population when compared with that reported by the autochthonous population. In the same sense, a recent study performed with the National Health Interview Survey of Denmark on an immigrant population with similar socio-demographic characteristics to residents of Spain, shows lower percentages of alcohol consumption in immigrants [30]. Also, other research carried out by Johnson *et al*. using data from the American National Health Survey indicate less probability of alcohol consumption for immigrants than for Americans [31]. In regard to smoking habits, immigrants residing in Spain smoke in less proportion than the autochthonous population, a situation that is also seen in other recent researches [32,33]. These circumstances are also seen in the Canadian study by Giorgiades *et al*. (the Ontario Health Survey) on factors that influence tobacco consumption where data show that 9.6% of immigrants are smokers compared to 17% of those born in Canada [34].

As for physical activity, immigrants register significantly lower levels, according to a study conducted by Dawson et al. in Sweden using data from the Swedish Survey of Living Conditions, in which the immigrant population reported engaging in lower levels of physical activity than the local Swedish population [35]. The negative consequences of these circumstances are evident and, it should not be forgotten that various studies have demonstrated the importance of socioeconomic factors (work overload, income) and cultural factors (less awareness about its beneficial effect) as determinant factors for physical activity [36].

Despite having a good health profile and claiming to have healthy lifestyles, immigrants residing in Spain during the time in which our study was performed, reported a more negative perception of their own health than the autochthonous people. The presence of language, cultural and administrative barriers etc. lead to social and economic deficiencies that produce greater vulnerability. These circumstances together with the lack of social and family support, xenophobia and other factors may contribute to this poor perception of health [26]. As such, it is necessary to delve deeper into this matter by performing more studies, including those based on qualitative methodology.

Although pathologies such as depression and anxiety are not significantly higher in the immigrant population than in the autochthonous population, we must not ignore the vulnerability of this group as different research [29,37] has alerted us of, which may be related to this poor perception of health.

When considering the results obtained for the use of healthcare resources by the immigrant population it must be noted that due to the characteristics of our National Health System, in Spain public healthcare is accessible to the immigrant population whether they have legal status or not. Theoretically a valid identity documents (healthcare card) is necessary to be attended yet the truth is that immigrants who lack this document and remain in the country as illegal aliens are also attended by the health system. It is important to note that in order to receive the healthcare card the only requisite is a registration certificate from the municipal register. In principle, we should not speak of difficulties in access to healthcare services by this group [38], but other authors have shown the difficulties and inequalities of access to healthcare services by immigrants in their studies, above all for those who do not have legal status [39,40].

The pattern of demand for healthcare resources by economic immigrants residing in Spain corresponds to the needs of a young population in good health condition which signifies less use of healthcare resources than the autochthonous population, as has been shown in different research at the national level [9,16,25] as well as research carried out in countries with similar healthcare systems to ours [13,41].

Nonetheless, our results show certain changes in the use of specific healthcare resources such as hospital emergency services, and detect a significant increase in the use of these services by the immigrant population in relation to other types of care. These circumstances have already recently been shown by other Spanish authors such as Rodriguez-Alvarez *et al*. with data from the Heath Survey of the Basque Country, showing significantly higher values than those obtained in our study, especially in Sub-Saharan and European non-EU immigrants [8]. Studies performed at the hospital level in Catalunya, based on registries, by Rue *et al*. coincide in showing this tendency [42]. This greater frequentation may be due to the ease of access and availability of hospital emergency services and lack of knowledge of the protocols to be followed to access healthcare services by the immigrant population, or to certain similarities in the immigrants' manner of accessing healthcare services in their native countries, but is not due to a worse health condition than the autochthonous population. "However, the possible existence of cultural, idiomatic and organizational barriers to primary health care services has to be taken into account."

Data on hospitalization of immigrants in Spain is still scarce. Studies carried out in our country by Sanz *et al [*24] or the study by Cots *et al*. [43] based on hospital discharges; conclude that consumption of hospital stays is significantly lower in the immigrant population than among the autochthonous population. Along the same lines, countries such as Canada, with data from the National Population Health Survey, have found hospitalization percentages that are practically equal when comparing the immigrant population with those born in Canada (11.4% vs. 11%) [44]. Also studies by Cacciani *et al*. performed in Italy with data from the Italian Hospital Information System [45] and the study in New York by Muennig [46], show lower hospitalization rates for foreigners.

Nonetheless, results from our study indicate a greater probability of hospitalization in the immigrant population when compared with the autochthonous population. These circumstances were already seen in the year 2003 when the National Health Survey, for the first time, included the immigrant population residing in the country [17]. For many authors, analysis of the total immigrant proportion is associated with a lower hospitalization rate, but when hospital admittance is analyzed by specific cause or by country of origin, some studies show greater values for hospitalization for the immigrant population [45]. The study by Muennig *et al*. carried out in the city of New York found that immigrants born in Sub-Saharan Africa have a greater probability of hospitalization (OR = 1,79 CI 95% 1,73–1,86) for the principle diagnostic categories, in relation to the autochthonous population [47]. In this sense, according to data from the Health Survey of the city of Madrid (Spain) in the year 2005, African immigrants who were hospitalized greatly surpassed the national average [48]. This difference in hospitalization may be determined by the age and the greater fertility rate of the immigrant population. Reasons for hospitalization, in this sense, are more frequently related with a young population in good health condition such as gynecological-obstetric or pediatric care [25,43,45]. In fact, there are clear indications of an increase in demand for medical services by immigrant women for reasons associated with reproduction [49] and actually 16.5% of children born in Spain have foreign mothers [50].

Finally, in regard to preventive measures, in a recent review, Fiscella proposed several potential explanations for the disparity in influenza vaccination among minorities: firstly, less frequent use of care due to access barriers; secondly, lower educational levels, inasmuch as education level is a strong predictor of receipt of preventive care; thirdly, patients' knowledge and attitudes towards the intervention might differ by race and ethnicity; fourthly, unconscious healthcare provider bias may affect delivery of care, so that a provider may be more likely to vaccinate a white rather than a minority-group patient; and lastly, minority patients may see providers who are less inclined to administer these vaccinations [51]. Although these explanations may be valid in Spain, more specific studies need to be performed.

The fact that the immigrant population residing in Spain visits a dentist significantly less frequently coincides with that shown in other studies where the number of visits to the dentist is not dependent on whether the individual is an immigrant or not, and is also not dependent on the country of origin or length of residency of these individuals [48]. These circumstances can be explained by the fact that, in Spain, oral health is a service not covered by public health. Research carried out by Tapias *et al*. to determine the influence of socio-demographic factors on the use of dental services in the pediatric population, shows less use of these resources among the population with lower economic incomes [52].

In reference to the limitations of the study, some of these make generalization of the conclusions, regarding the results obtained, difficult. A significant limitation of the health surveys comes from or arises from the use of self-reported data, and as such we cannot forget that some response may be socially conditioned in both study populations.

Another limitation is the inclusion of the new countries recently incorporated to European Union. These are countries that are economically less favoured than Spain, although it is difficult to quantify this influence, as both Bulgaria and Romania were incorporated on January 1^st^, 2007. Therefore, individuals having these nationalities were not considered EU-citizens for a period of time while the study was being carried out.

Future investigation will need to consider the differences in health care utilization and health characteristics between member states that may influence demands for service. But in the 2006 survey the proportion of migrants from the EU, Canada and the USA was very small and in a preliminary analysis (age adjusted) we found a similar health profile and use of health services to the indigenous population so we decided to analyze them as a single group.

We can explain this circumstance if we bear in mind that the immigrant population, coming mainly from European Union countries, tends to be made up of people who are establishing their final residence in our country [53].

It should be pointed out that the Spanish National Health Survey has not been validated for chronic illnesses or lifestyles. It has only been validated for the obesity variable from which it can be concluded that self-reported data is an efficient way of obtaining information about body max index (BMI) but it tends to slightly underestimate BMI and the proportion of participants with elevated BMI [54].

Not knowing exactly the use of hospital services by economic immigrants we cannot precisely analyze its frequency or its usage profile. We must take into account the fact that certain characteristics associated with a long period of stay in Spain have evolved towards the patterns of the autochthonous population and as such, the differences in behaviour of the recent immigrant group are underestimated [55]. Actually the *duration of residence in Spain *variable, which unfortunately is not currently included in the ENSE06, will be useful in order to more precisely determine immigrants' use of health resources including ER.

Lastly, the initial response rate to the SNHS was 65%, so the existence of possible non-response bias should thus be considered as this rate is influenced by the country of origin variable [56]. This analysis has included the assessment of access to accommodations and the lack thereof. The percentage of inability to answer has also been taken into account. These differences can probably be explained by the lack of Spanish-language knowledge of certain EU members and non-EU members.

The fact that the questionnaire was written in Spanish makes it easier for the Latin American population, as well as for immigrants who have been in Spain for a long time, to answer the questions. Therefore, over representation of the Latin American population is likely to occur. **This over representation of the Latin American population may constitute a possible bias in the ED utilization, lifestyles and other important outcome variables of the study, so the results for the entire immigrant population may be biased by this subgroup**

We can not forget that in year 2004 there was a Regularization Process in Spain and around 800.000 immigrants previously considered illegal received residence cards. We think that the non response bias is possible smaller in SNHS06 than SNHS03 due to the legal situation of a much greater proportion of immigrants.

## Conclusion

Among the principal conclusions of the study, the fact that in Spain the immigrant population is in excess of 11% of the population must be emphasized as this is transforming the structure of the national population. This fact may have important repercussions on the health of the population because even though the foreign population interviewed (economic immigrants) have better parameters in relation to lifestyles, they consume less alcohol, smoke less, and less physical activity; they have a poor perception of their health nonetheless. Despite the fact that immigrant population shows higher percentages of emergency attendance and hospitalization than the indigenous population, with respect to the use of healthcare resources, their usage of healthcare resources such as drugs, influenza vaccinations or visits to the dentist is lower.

In this sense, knowing the length of residency in the country is key to determining health conditions and patterns of use of healthcare resources.

As such, the needs in the healthcare environment of immigrants are comparable to those of the autochthonous population without ignoring the effect known as the healthy immigrant effect. The social and economic deficiencies may create barriers and inequalities that increase the vulnerability of the immigrant population with measures for social inclusion and improvement of the living conditions of the least-favoured immigrants being necessary. New research, dynamic in nature, continues to be necessary to facilitate studies on health trajectories of the immigrant population and patterns of demand for healthcare.

## Competing interests

The authors declare that they have no competing interests.

## Authors' contributions

PCG and RJG conceived of the study, have written the document, and supervised all aspect of its implementation. VHB has analyzed the information, ALA and AGM, helps with the reading and review. All authors helped to conceptualize ideas, interpret findings, and review of the manuscript.

## Pre-publication history

The pre-publication history for this paper can be accessed here:


